# *Phytophthora cinnamomi* populations collected from avocado in the United States exhibit high adaptive capacity to climate and disease control methods

**DOI:** 10.3389/fpls.2026.1838248

**Published:** 2026-06-22

**Authors:** Benjamin K. Hoyt, Savannah Salas, Jonathan H. Crane, Monica Navia-Urrutia, Romina Gazis, Liliana M. Cano, Achyut Adhikari, Miaoying Tian, John Jifon, Ricardo Goenaga, Luz M. Serrato-Diaz, James E. Adaskaveg, Patricia Manosalva

**Affiliations:** 1 Department of Microbiology and Plant Pathology, University of California, Riverside, Riverside, CA, United States; 2 Tropical Research and Education Center, Institute of Food and Agricultural Sciences, University of Florida, Homestead, FL, United States; 3 Department of Plant Pathology, Indian Research and Education Center, Institute of Food and Agricultural Sciences, University of Florida, Fort Pierce, FL, United States; 4 Department of Plant and Environmental Protection Sciences, University of Hawai‘iat Mānoa, Honolulu, HI, United States; 5 Department of Horticultural Sciences, Texas A&M AgriLife Research, Wescalo, TX, United States; 6 USDA, Agricultural Research Service (ARS)-Tropical Agriculture Research Station, Mayaguez, Puerto Rico

**Keywords:** fungicide resistance, oomycete, Phytophthora root rot, potassium phosphite, virulence

## Abstract

*Phytophthora cinnamomi*, the causal agent of Phytophthora root rot (PRR), poses a persistent threat to the United States avocado industry, the top domestic producer and consumer. Avocado growers are facing clonal A2 *P. cinnamomi* populations challenging their current PRR control methods. In this study, we characterized 125 isolates collected from orchards in California, Florida, Hawaii, Texas, and Puerto Rico for radial growth per day, optimal growth temperature, *in vitro* fungicide sensitivity, and virulence on D’Anjou pear fruit and UC2001 avocado seedlings. Across all isolates, optimal growth occurred most frequently at a range from 22 to 25 °C; however, a subset of isolates from Hawaii, Florida, and California exhibited higher optimal growth temperatures (28˚C and 30˚C) suggesting thermal adaptation in warmer regions. Potassium phosphite EC_50_ values spanned from 4.61 to 763.13 µg/ml, with significantly higher insensitivity in isolates from California and Florida, reflecting the continued overuse of this fungicide in these major production states. In contrast, baseline sensitivities to ethaboxam, mandipropamid, mefenoxam, fluopicolide, and oxathiapiprolin were uniformly high, with narrow, unimodal EC_50_ distributions across states. Finally, a wide range of virulence among isolates was detected using avocado seedlings and D’Anjou pear fruits with isolates from California and Puerto Rico being the most virulent. Together, this data documents extensive phenotypic diversity within clonal A2 *P. cinnamomi* populations including heat−adapted and phosphite−insensitive lineages, establishes multi−state fungicide sensitivity baselines, and underscores the need for continued surveillance, integrated fungicide stewardship (especially phosphonates), and rootstock screening against phenotypically diverse populations to sustain avocado PRR management and ensure the United States’ avocado industry sustainability and profitability.

## 1 Introduction

Avocado (*Persea americana*, Mill) is a major fruit tree crop produced worldwide. The avocado fruit is a globally popular, nutrient-dense superfood with numerous health benefits (Dreher and Davenport, 2013). In 2023, the global avocado production reached 10 million metric tons with Mexico being the top producer and exporter (FAO, 2025). *Phytophthora cinnamomi*, the causal agent of avocado Phytophthora root rot (PRR), is a highly invasive soilborne oomycete pathogen recognized as a major threat for agriculture as well as global biodiversity (Burgess et al., 2017). Its exceptionally broad host range, infecting more than 5, 000 plant species, has led to its inclusion among the world’s top 100 invasive alien species (Lowe et al., 2000). Because PRR is one of the most destructive diseases affecting the sustainability of the avocado industry, it has been the focus of extensive research in major producing regions including the United States (Belisle et al., 2019a; Crandall, 1948; Shands et al., 2025; Zentmyer, 1976), Mexico (Mondragón-Flores et al., 2022; Ochoa-Fuentes et al., 2007; Zentmyer, 1951), and South Africa (Engelbrectht et al., 2022; Köhne, 1998; Wager, 1942). As global avocado cultivation has expanded to every continent except Antarctica, reports of PRR outbreaks and studies on *P. cinnamomi* have increased markedly over the last decade (Kavroulakis et al., 2024; Kurbetli et al., 2020; Madhu et al., 2025; Ramírez-Gil et al., 2021).

The United States is one of the world’s largest avocado consumers and its top importer (FAO, 2025; Hammami et al., 2024). Domestically, California (CA) remains the leading producer, followed by Florida and Hawaii (Hammami et al., 2024). PRR causes substantial economic losses through reduced yields, diminished fruit quality, and the need for costly disease management practices (Coffey, 1987; Ramírez-Gil et al., 2017). Disease severity is further intensified by environmental conditions common in U.S. producing states, including waterlogged soils, increased hurricane frequency and flooding in Florida and Puerto Rico, heavy rainfall in Hawaii, and widespread pathogen presence in Texas orchards; all factors that constrain the industry expansion in the United States (Bender et al., 2012; Hawaii Department of Agriculture, 2024; Kunta et al., 2007; Ploetz and Schaffer, 1989; RoyChowdhury et al., 2016).

*Phytophthora cinnamomi* is a heterothallic hemibiotrophic pathogen requiring two mating types (A1 and A2) for sexual reproduction; however, only clonal A2 populations have been associated with avocado PRR worldwide (Calle-Henao et al., 2020; Dobrowolski et al., 2003; Engelbrecht et al., 2022; Hüberli et al., 2001; López-Herrera and Pérez-Jiménez, 1995; Shands et al., 2025). The pathogen reproduces primarily through its asexual cycle: sporangia release motile zoospores that move through moist or waterlogged toward avocado roots by chemotaxis (Hardham and Blackman, 2018). Under unfavorable conditions, thick-walled chlamydospores allow this pathogen long-term survival (Jung et al., 2013). In orchards, *P. cinnamomi* spreads through soil, water, and infested plant material, while human-mediated movement of contaminated soil, nursery stock, or equipment enables long-distance dispersal (Dreistadt, 2007). Infections of feeder roots, and occasionally trunk cankers, lead to canopy decline, reduced yields, and often tree mortality (Hardham and Blackman, 2018; Ploetz and Parrado, 1987). Entirely newly planted orchards may fail in the absence of effective disease management (Faber et al., 2012). Current PRR management includes chemical treatments (e.g., phosphonates, mefenoxam, and oxathiapiprolin) (Adaskaveg et al., 2024), tolerant or resistant rootstocks (Bender et al., 2012; Crane et al., 2024), accurate diagnostics (Wilkins et al., 2025), and cultural practices such as mulching and irrigation management (Ramírez-Gil and Morales-Osorio, 2020). However, reports of *P. cinnamomi* isolates exhibiting reduced sensitivity to potassium phosphite and increased virulence on widely used avocado rootstocks such as Dusa^®^ and Duke7 have risen in the past decade (Andronis et al., 2024; Belisle et al., 2019a, b; Dobrowolski et al., 2008; Hoyt et al., 2026; Hunter et al., 2023; Ma and McLeod, 2014; Sánchez-González et al., 2019; Shands et al., 2025). These findings align with the substantial phenotypic diversity documented in *P. cinnamomi* populations from avocado orchards in Mexico (Mondragón-Flores et al., 2022; Ochoa-Fuentes et al., 2007), Colombia (Calle-Henao et al., 2020; Palacios-Joya et al., 2025), New Zealand (Hunter et al., 2023), Australia (Wilkinson et al., 2001), and South Africa (Ma and McLeod, 2014).

In California, our previous work identified two clonal A2 lineages associated with PRR, both displaying broad phenotypic variation in radial growth rate, optimal growth temperature, fungicide sensitivity, and virulence (Belisle et al., 2019a, b; Shands et al., 2025). Pathogen isolates collected from southern growing regions exhibited less sensitivity to higher growth temperatures and potassium phosphite but at the same time exhibited more virulence when infecting commonly used avocado rootstocks (Belisle et al., 2019a, b; Shands et al., 2025). Moreover, we found that *P. cinnamomi* isolates corresponding to one of the A2 clonal lineages detected only in Southern California growing regions (Pagliaccia et al., 2013) were genetically similar to *P. cinnamomi* populations collected from avocado orchards in Mexico (Mexican origin) (Ochoa-Fuentes et al., 2015) suggesting migration between the two regions (Shands et al., 2025). Despite the importance of avocado PRR to the U.S. avocado production, comprehensive genotypic and phenotypic characterization of *P. cinnamomi* in other producing states remains limited. To address this gap, we expanded the phenotypic characterization of *P. cinnamomi* populations from avocado orchards in Florida, Hawaii, Texas, and Puerto Rico, examining *in vitro* growth rates, optimal growth temperatures, *in vitro* fungicide sensitivity, and virulence. We also evaluated phenotypic shifts in the current California populations. Continued monitoring of domestic pathogen populations is essential to guide the development, evaluation, and long-term durability of PRR management strategies, ultimately supporting the sustainability, competitivity, and profitability of the U.S. avocado industry.

## 2 Materials and methods

### 2.1 Collection of *Phytophthora cinnamomi* isolates

*Phytophthora cinnamomi* isolates from California, Florida, Hawaii, Texas, and Puerto Rico (2020-2022) were recovered from symptomatic avocado trees and soil samples using multiple isolation methods. Root and soil plating were performed as described by Belisle et al. (2019a), and soil baiting following the protocols of Hao et al. (2018) using pear fruits or avocado leaves. Isolates were identified morphologically, and single-zoospore cultures were generated following Lonsdale et al. (1988). Molecular confirmation was conducted by Sanger sequencing of the internal transcribed spacer (ITS) region and a *P. cinnamomi*-specific TaqMan quantitative PCR assay targeting the mitochondrial *atp9*-*nad9* locus (Bilodeau et al., 2014; Belisle et al., 2019a; Miles et al., 2017). In addition, four isolates collected from diseased walnut trees in California were provided by Dr. Gregory T. Browne (University of California, Davis) (Browne et al., 2015). All isolates were stored as colonized 10% clarified V8 (V8C) agar plugs in water (Ko, 2003) until use.

### 2.2 *In vitro* radial growth rate and optimal growth temperature

Isolates were assessed for *in vitro* radial growth rate per day (RGPD) and optimal growth temperature on 10% V8C agar at 22˚C, 25˚C, and 28˚C following Shands et al. (2025). For isolates that continued to increase in growth at 28˚C, additional measurements were obtained at 30˚C and 32˚C. The previously characterized isolate, Pc2113, was included as an internal control to ensure the growth rate assay yielded similar values to those previously reported (Shands et al., 2025). Experiments were performed in triplicate and repeated twice using all isolates.

### 2.3 *In vitro* sensitivity to Oomycota fungicides

Sensitivity to potassium phosphite (Prophyt, 34.3% phosphorous acid [FRAC code P07]; Helena Chemical Co., Collierville, TN) was conducted using the agar dilution method described in Adaskaveg et al. (2015) with potassium phosphite concentrations of 0 (control), 5, 25, 100, 150, 300, 600, or 1000 μg/ml. EC_50_ values (inhibition of 50% mycelial growth) were calculated as described in Shands et al. (2025).This experiment was performed in triplicate and repeated twice. *In vitro* sensitivity to mefenoxam (Ridomil Gold^®^ SL [FRAC code 4]; Syngenta Crop Protection, Greensboro, NC), oxathiapiprolin (Orondis^®^ [FRAC code 49]; Syngenta Crop Protection, Greensboro, NC), mandipropamid (Revus [FRAC code 40]; Syngenta Crop Protection, Greensboro, NC), fluopicolide (Presidio [FRAC code 43]; Valent U.S.A., Walnut Creek, CA), and ethaboxam (Elumin [FRAC code 22]; Valent U.S.A., Walnut Creek, CA) was done using the spiral gradient dilution method (SGD) described by Förster et al. (2004) with modifications outlined in Belisle et al. (2019b). Aqueous stock solutions of mefenoxam (50 µg/ml), oxathiapiprolin (1 µg/ml), mandipropamid (10 µg/ml), fluopicolide (100 µg/ml), and ethaboxam (50 µg/ml) were applied to 15-cm 10% V8C agar plates using a spiral plater (Eddy Jet 2W, Neutec Group, Inc., Farmingdale, NY) in exponential deposition mode. Sterile Milli-Q water (Millipore Sigma, Burlington, MA, USA) was used for control plates. Two mycelium-covered cellophane strips per isolate were placed opposite each other on each chemical- or water-amended plates and incubated at 22 °C for 2 days. The point of 50% growth inhibition was recorded and EC_50_ values were calculated using the Spiral Gradient Endpoint (SGE) software (Spiral Biotech). The previously characterized isolate, Pc2113, was included as an internal control to ensure the assay yielded EC_50_ values similar to those previously reported (Belisle et al., 2019b; Shands et al., 2025). Experiments were repeated twice using all isolates.

### 2.4 Virulence assays

Virulence was assessed on D’Anjou pear fruit as described by Shands et al. (2025). Six fruits per isolate were inoculated with 20 µl of a 1x10^4^ zoospore/ml suspension. Lesion area was recorded at 3-, 4-, and 5-days post inoculation (DPI) to calculate area under the disease progression curve (AUDPC). Virulence on avocado was evaluated using UC2001 seedlings (Menge et al., 1999) following Shands et al. (2025) with modifications. Avocado seedlings were grown for 12–14 weeks at temperatures and humidity conditions ranging from 22˚C to 30˚C and 35% to 72%, respectively. For each isolate, five seedlings were inoculated with 3.4 g of *P. cinnamomi*-colonized millet, and the percentage of diseased roots was assessed at 5 weeks post-inoculation (WPI). Isolates that were previously characterized, Pc2113 and Pc2109, served as internal controls to ensure virulence assays yielded similar values to those previously reported (Belisle et al., 2019b; Shands et al., 2025). Seedlings treated with uninoculated millet served as negative controls. The experiment was conducted twice using pear fruits and once using UC2001 avocado seedlings for all isolates.

### 2.5 Statistical analysis

Data normality was assessed for using the Shapiro-Wilk test. Except for seedling virulence assays, all datasets were analyzed using generalized linear mixed models (GLMMs) with a gamma distribution, log link function, and experimental repeats as random effects. Seedling virulence data were analyzed using a generalized linear model (GLM). Least squares means were calculated using LSMEANS with a TUKEY adjustment. Analyses were performed in R v.4.3.2 (R Core Team, 2023) using the lme4 (Bates et al., 2015) and lsmeans (Lenth, 2016) packages. Results were considered significant at *P* ≤ 0.05.

## 3 Results

### 3.1 *P. cinnamomi* populations from California, Florida, and Hawaii exhibit adaptation to higher temperatures

A total of 125 P*. cinnamomi* isolates were examined in this study, including four isolates from walnut in California. Avocado isolates originated from California (n = 34), Florida (n = 25), Hawaii (n = 12), Texas (n = 25), and Puerto Rico (n = 25) (**Table 1**). The RGPD values for all isolates at each growth temperature tested are summarized in **Supplementary Table 1**. Across all isolates, optimal growth temperatures occurred at 22 °C (52.8%) and25 °C (31.2%), while only 13% growing optimally at 28 °C. Previously, wereported that the *P. cinnamomi* population (1994-2019) from southern growing areas in California (San Diego and Riverside Counties [CA-South]) displayed greater virulence and enhanced growth at warm temperatures compared to isolates from northern growing areas (Ventura and Santa Barbara [CA-North]) (Shands et al., 2025). Here, we observed similar patterns in the current population (2020-2022). CA-North isolates grew significantly faster than CA-South isolates at 22 °C and 25 °C, but not at 28 °C. Consistent with this, 53% and 47% of CA-North isolates exhibited optimal growth temperatures of 22 °C and 25 °C, respectively, whereas 41% of CA-South isolates grew optimally at 28 °C and 29% at 22 °C and 25 °C. The average RGPD of CA-North isolates decreased significantly at 28 °C compared with 22 °C and 25 °C, while CA-South isolates maintained similar growth across temperatures (**Supplementary Table 2** and **Supplementary Figure 1**).

**Table 1:** Sample information of all *Phytophthora cinnamomi* isolates used in this study.

Number of isolates	State	County	Host	Year collected (range)
7	California	Riverside	Avocado	2020-2022
10	California	San Diego	Avocado	2020-2022
12	California	Santa Barbara	Avocado	2020-2022
5	California	Ventura	Avocado	2020-2022
9	Florida	Miami-Dade	Avocado	2020-2021
11	Florida	Lee	Avocado	2022
2	Florida	St. Lucie	Avocado	2022
3	Florida	Polk	Avocado	2022
2	Hawaii	Honolulu	Avocado	2021
7	Hawaii	Hawaii	Avocado	2021-2022
3	Hawaii	Kauai	Avocado	2022
7	Texas	Cameron	Avocado	2021
18	Texas	Hidalgo	Avocado	2022
9	Puerto Rico	Adjuntas	Avocado	2021-2022
16	Puerto Rico	Isabela	Avocado	2022
1	California	Colusa	Walnut	2010
1	California	Merced	Walnut	1996
2	California	San Joaquin	Walnut	2009

**Figure 1:**
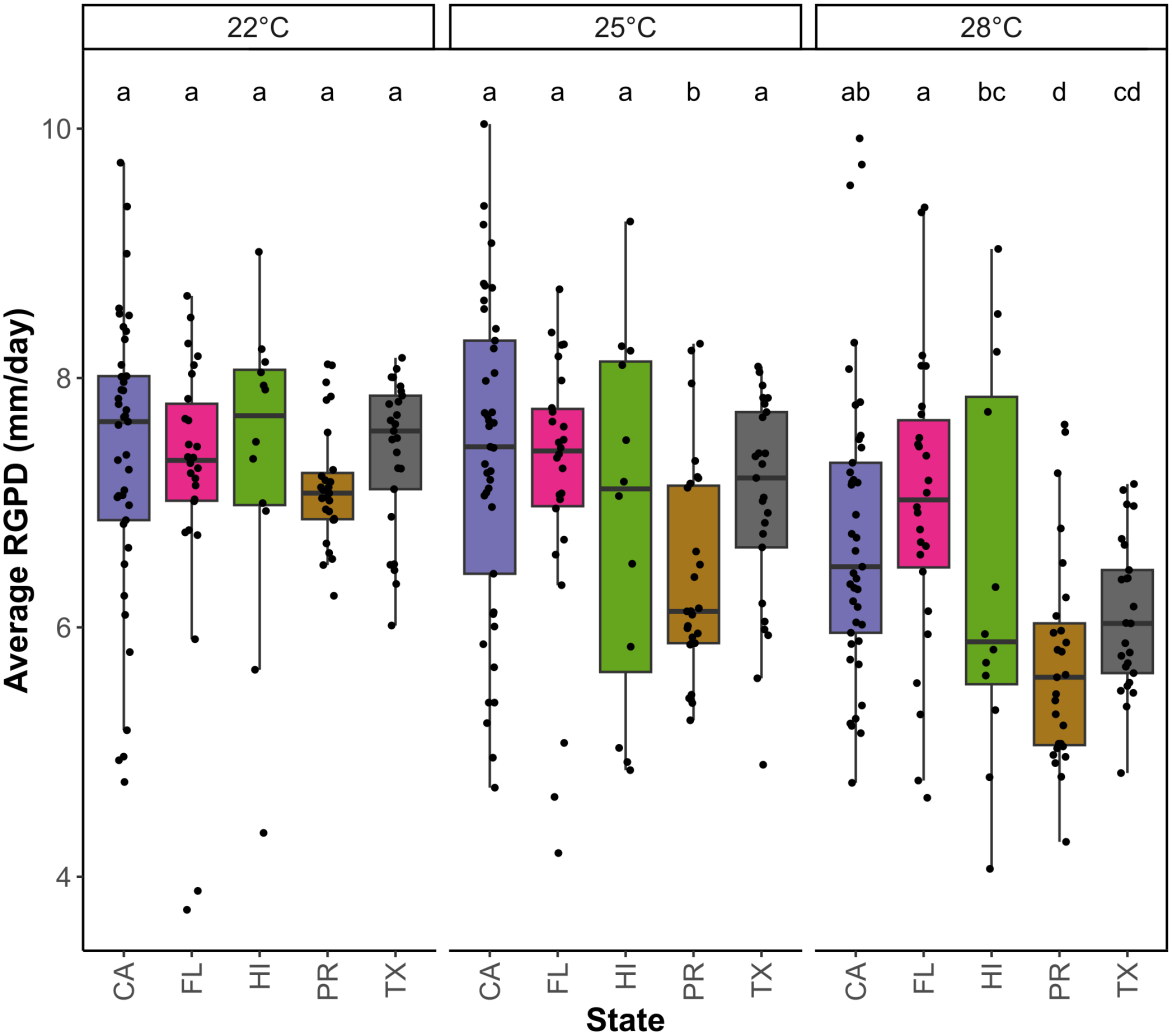
Phenotypic variability of *Phytophthora cinnamomi* isolates collected from major avocado growing regions in the United States regarding their *in vitro* radial growth per day (RGPD) at 22°C, 25°C, and 28°C. Boxplots display the median RGPD and distribution of data of *P. cinnamomi* isolates at each temperature for each state. Bars followed by the same letter do not differ significantly in average RGPD between states within each temperature, according to the least squared means test at *P* < 0.05. California = CA, Florida = FL, Hawaii = HI, Puerto Rico = PR, and Texas = TX.

**Figure 2:**
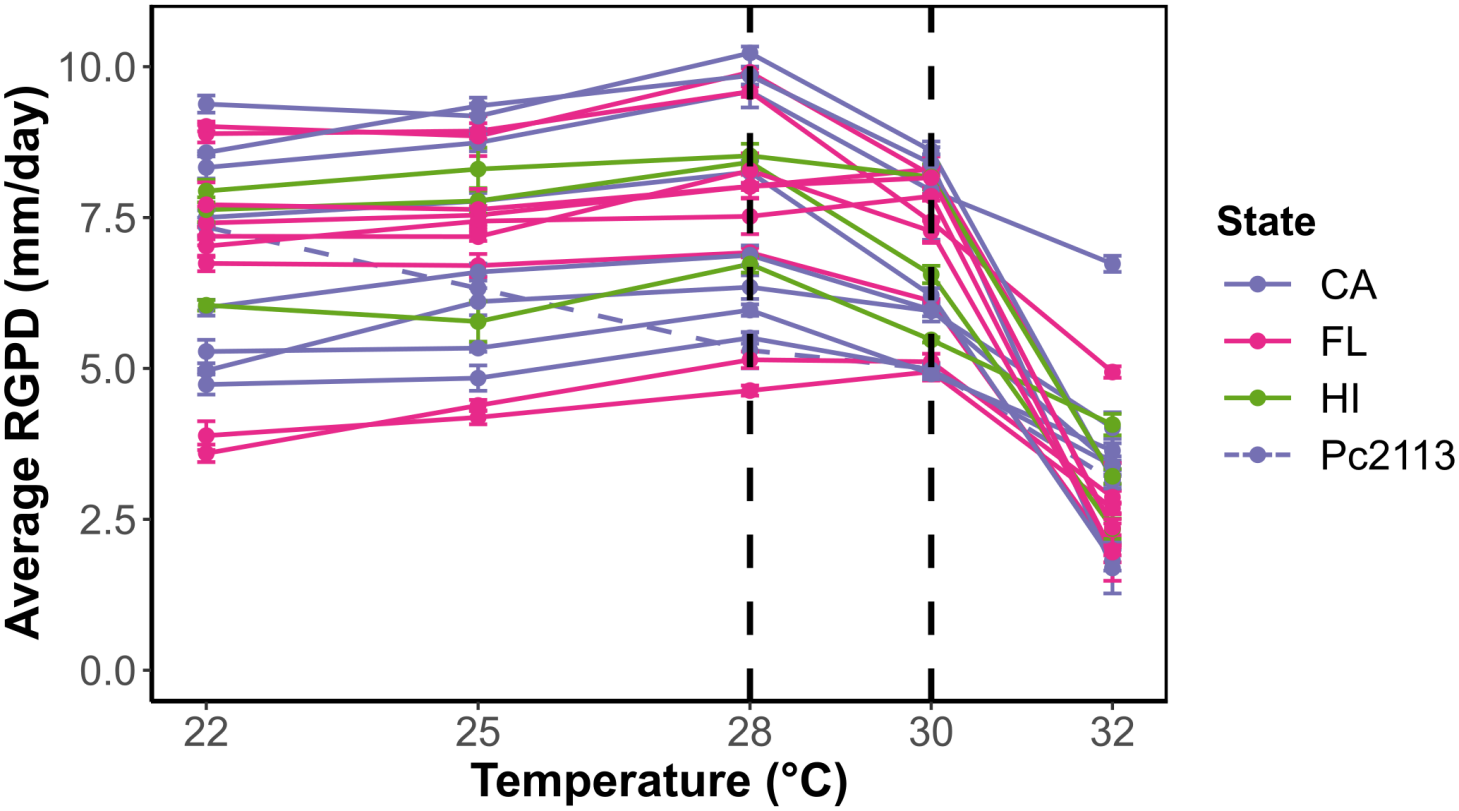
Assessment of *in vitro* radial growth per day (RGPD) and optimal growth temperature of the subset of *Phytophthora cinnamomi* isolates exhibiting no growth reduction at 25°C. Isolates were tested for their RGPD at 22°C, 25°C, 28°C, 30°C and 32°C. Optimal growth temperatures are indicated as dashed vertical black lines as well as error bars. Pc2113 isolate was used as internal control and is indicated as dashed purple line. California = CA, Florida = FL, Hawaii = HI, Puerto Rico = PR, and Texas = TX.

Significant isolate-level differences in RGPD were detected at all temperatures. As expected, control isolate Pc2113 grew optimally at 22°C. The slowest-growing isolates at 22 °C and 25 °C were from Florida (AVR-001 and AVR-008), and at 28 °C from Hawaii (HI-8-SZ1). All fastest-growing isolates originated from California (**Figure 1**; **Supplementary Table 1**). Significant differences in average RGPD among states were detected at 25 °C and 28 °C, with isolates from Puerto Rico exhibiting the lowest values compared with those from California, Florida, and Hawaii (**Figure 1**; **Supplementary Table 3**). Most isolates from Texas (76%) and Puerto Rico (80%) had an optimal growth at 22°C, and none grew optimally above 25°C. Twenty isolates from California, Florida, and Hawaii did not exhibit reduced growth at 28°C; prompting further evaluation at 30 °C and 32 °C. Of these 20 isolates, 16 grew best at 28°C. Seven of eight isolates from California with optimal growth at 28°C originated from CA-South (Riverside and San Diego) while four of nine Florida isolates grew optimally at 30°C (**Table 2** and **Figure 2**). These results highlight the adaptive capacity of *P. cinnamomi* populations to higher temperatures in Southern California growing regions, Florida, and Hawaii.

**Table 2:** Optimal growth temperature of a subset of *Phytophthora cinnamomi* isolates that did not exhibit reduction in their *in vitro* radial growth per day (RGPD) at 25°C.

Isolate ID	State	Optimal growth temperature (°C)	Average RGPD (mm/day)
Pc2113	California	22**	7.34 ± 0.28
578	California	28*	9.59 ± 1.29
647	California	28*	6.35 ± 0.34
FG 24-2	California	28*	5.51 ± 0.17
FG 29-1	California	28*	5.97 ± 0.16
FG 57-2	California	28*	6.88 ± 0.28
FG 90-2	California	28*	8.25 ± 0.33
Gary 1	California	28*	9.85 ± 0.26
Gary 2	California	28*	10.23 ± 0.58
AVR-001	Florida	28*	5.14 ± 0.24
AVR-008	Florida	30*	4.95 ± 0.26
F13-2	Florida	28*	6.92 ± 0.14
FL 2-1	Florida	28*	9.59 ± 0.15
FL 2-2	Florida	28*	9.91 ± 0.12
FL 4-1	Florida	30*	8.30 ± 0.07
R23 T3-4	Florida	30*	8.16 ± 0.30
R23 T3-5	Florida	30*	7.85 ± 0.60
R8 T1-1	Florida	28*	8.28 ± 0.49
HI-1-SZ1	Hawaii	28*	8.52 ± 0.34
HI-14-SZ1	Hawaii	28*	6.73 ± 0.25
HI-2-SZ1	Hawaii	28*	8.42 ± 0.23

These isolates were further assayed at higher temperatures to calculate their optimal growth temperature. Optimal growth temperature, average RGPD values, and standard deviation are displayed for each isolate.

Average RGPD was significantly different at the optimal growth temperature (temperature with the highest RGPD value) when compared with the values at the immediately lower* and higher** temperature tested (P < 0.05).

### 3.2 Isolates from California and Florida exhibit reduced sensitivity to potassium phosphite

**Figure 3:**
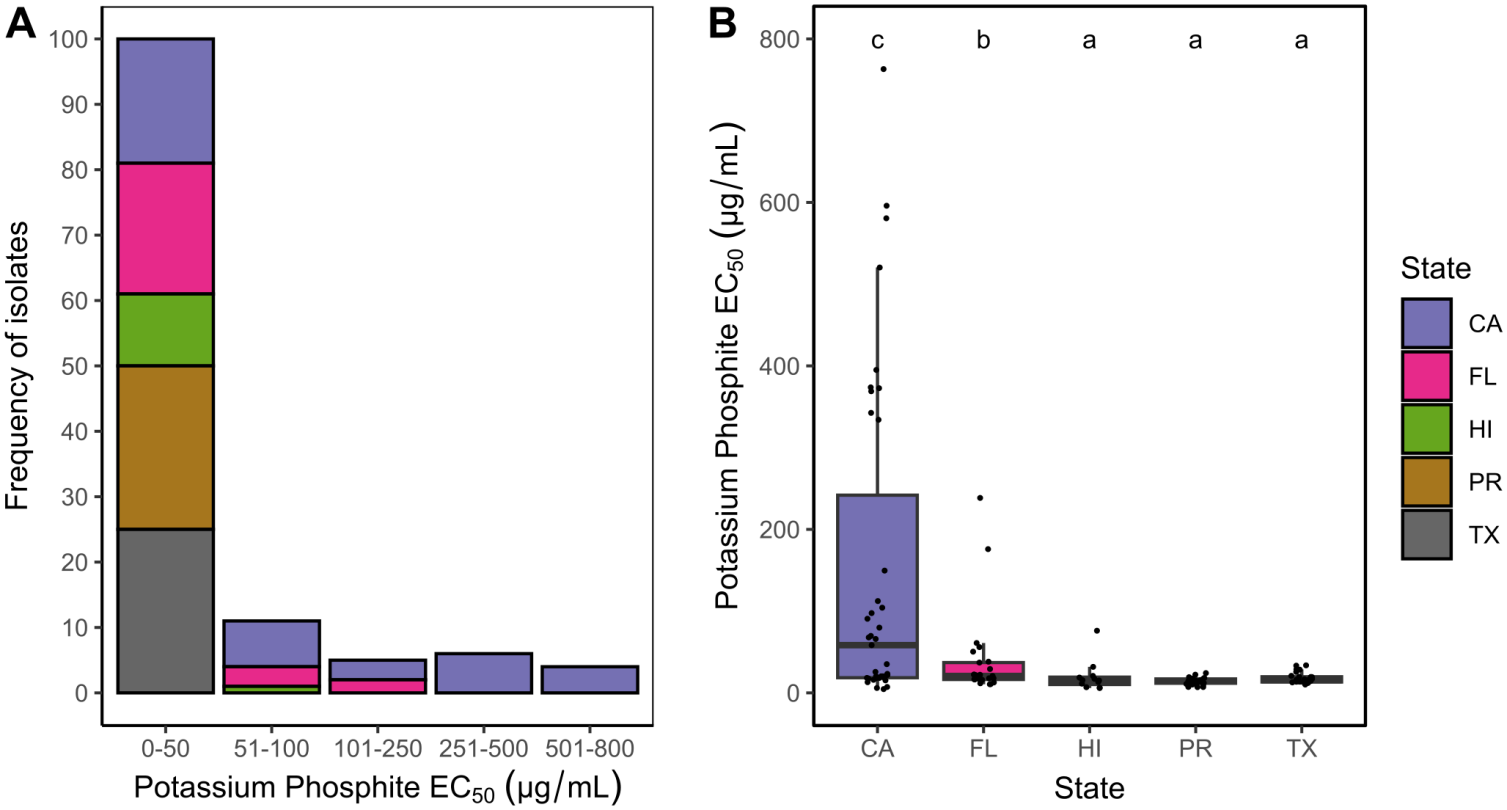
*In vitro* potassium phosphite sensitivity of *Phytophthora cinnamomi* isolates. **(A)** Frequency histograms of EC_50_ values (µg/ml) of 125 isolates. Bar heights indicate the number of isolates within each bin. Bin widths were assigned based on distributions of potassium phosphite sensitivities previously reported for *P. cinnamomi* isolates from avocado in California (Belisle et al., 2019b; Shands et al., 2025). **(B)** Boxplots showing the median and distribution of *in vitro P. cinnamomi* potassium phosphite EC_50_ values (µg/ml) for each state. Bars followed by the same letter did not significantly differ in the average EC_50_ value, according to the least squared means test at *P* < 0.05. Bars are color-coded based on the geographic origin of isolates (CA = California, FL = Florida, HI = Hawaii, PR = Puerto Rico, and TX = Texas).

All isolates were evaluated for their *in vitro* potassium phosphite sensitivity measured as EC_50_ (µg/ml) values which ranged from 4.61 (Gary 2 from CA) to 763.13 µg/ml (592 from CA). Significant differences were detected both within and among states (**Figure 3**; **Supplementary Table 1**). California isolates exhibited the greatest EC_50_ range, followed by isolates from Florida (10.55 to 238.51 µg/ml), Hawaii (5.93 to 76.04 µg/ml), Texas (10.17 to 33.67 µg/ml), and Puerto Rico (7.01 to 22.29 µg/ml) (**Figure 3**; **Supplementary Table 1**). These EC_50_ values were distributed into five bins with EC_50_ values <50 µg/ml (n = 100), 51-100 µg/ml (n = 11), 101-250 µg/ml (n = 5), values of 251-500 µg/ml (n = 5), and 501-800 µg/ml (n = 4). Based on previous studies reporting potassium phosphite *in vitro* sensitivities for *Phytophthora* spp. including *P. cinnamomi*, we classified isolates with EC_50_ values < 50 µg/ml as highly sensitive, < 100 µg/ml as moderate sensitive, and > 100 µg/ml as insensitive isolates (Belisle et al., 2019a; Hao et al., 2021; Shands et al., 2025). The frequency distribution of potassium phosphite EC_50_ values was unimodal and predominantly skewed toward high sensitivity (< 50 µg/ml) (**Figure 3A**). Average EC_50_ values for isolates from Puerto Rico, Texas, and Hawaii (all < 100 µg/ml) were significantly different from those of isolates from Florida and California (**Figure 3B**; **Supplementary Table 3**). Interestingly, only isolates from California and Florida showed high insensitivity to potassium phosphite (EC_50_ values >100 µg/ml). California isolates were amongst the most insensitive isolates exhibiting values over 500 µg/ml (**Figure 3**; **Supplementary Table 1**). These patterns suggest a strong capacity for resistance development in pathogen populations affecting the major avocado producers and where potassium phosphite is heavily used.

### 3.3 Baseline sensitivities to Oomycota fungicides across U.S. avocado producing regions

**Figure 4:**
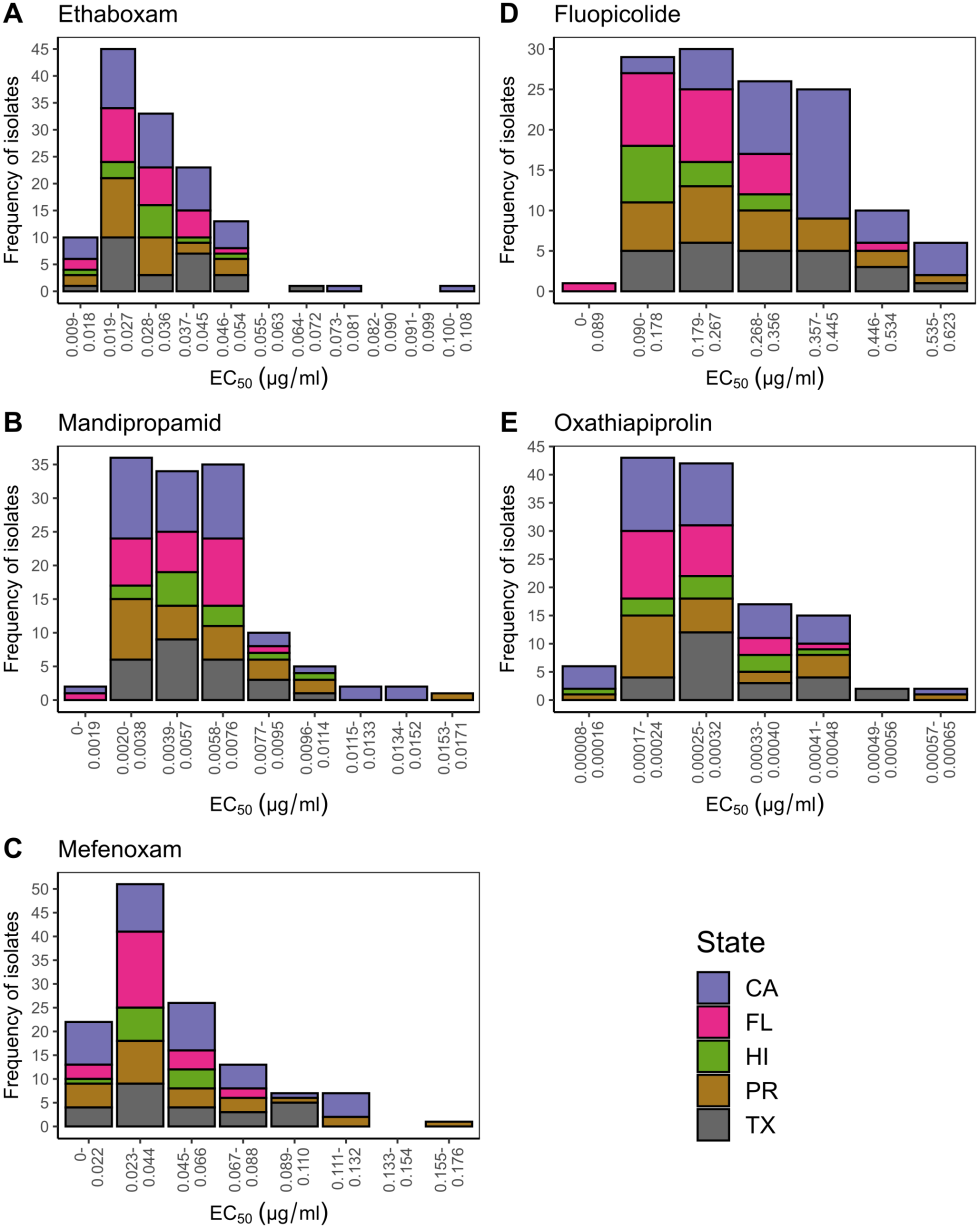
Frequency histograms of *in vitro* fungicide sensitivities (µg/ml) of *P. cinnamomi* isolates to ethaboxam **(A)**, mandipropamid **(B)**, mefenoxam **(C)**, fluopicolide **(D)**, and oxathiapiprolin **(E)** collected from different avocado producing states in the United States. Bar heights indicate the number of isolates within each bin. Bin widths were calculated based on Scott’s rule (1979). Bars are color-coded based on the geographic origin of isolates (CA = California, FL = Florida, HI = Hawaii, PR = Puerto Rico, and TX = Texas).

**Figure 5:**
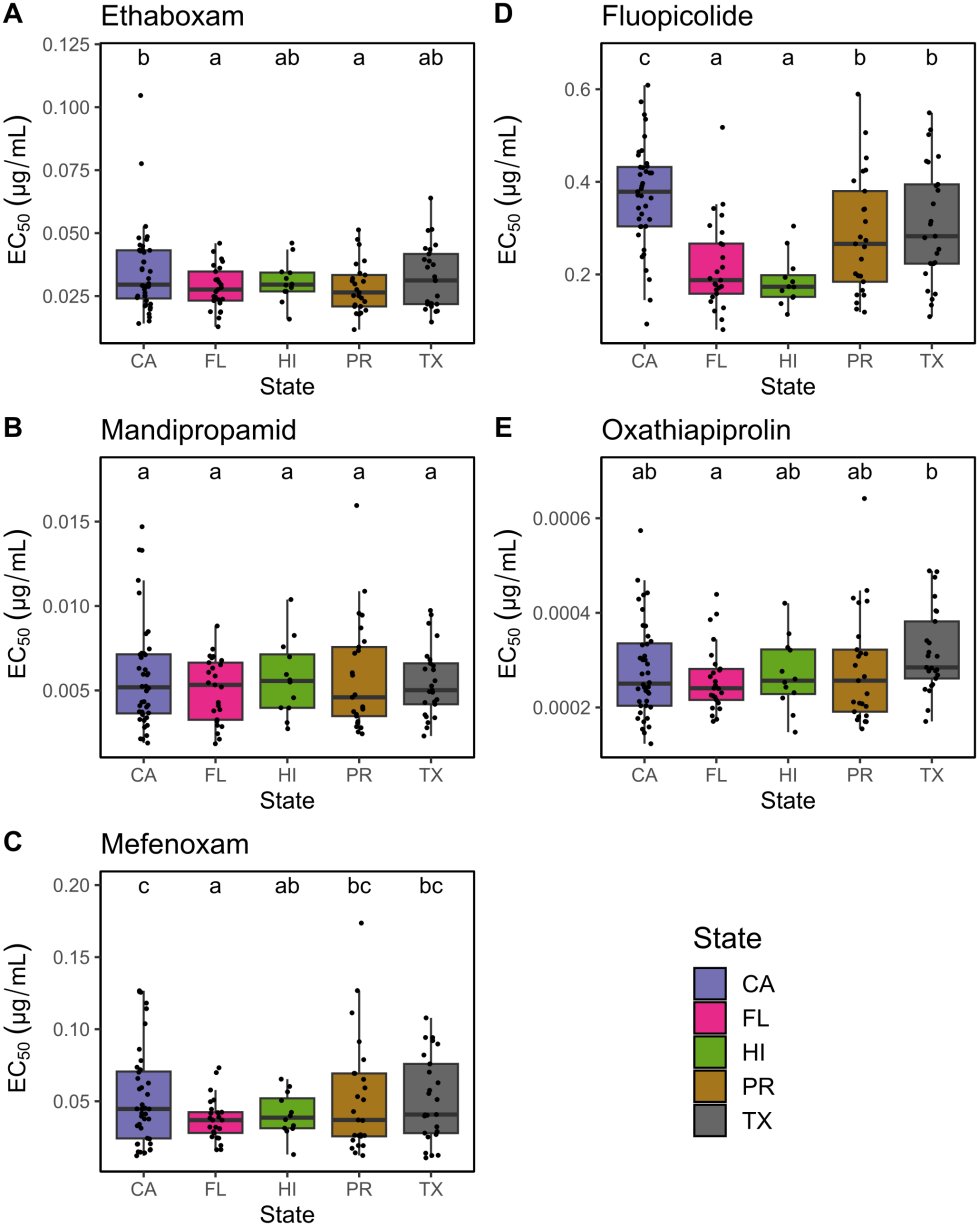
*In vitro* sensitivity of *Phytophthora cinnamomi* isolates to fungicides. Boxplots showing the median and distribution of the EC_50_ values (µg/ml) for ethaboxam **(A)**, mandipropamid **(B)**, mefenoxam **(C)**, fluopicolide **(D)**, and oxathiapiprolin **(E)**. Bars followed by the same letter did not significantly differ in the average EC_50_ value, according to the least squared means test at *P* < 0.05. Bars are color-coded based on the geographic origin of isolates (CA = California, FL = Florida, HI = Hawaii, PR = Puerto Rico, and TX = Texas).

To establish baseline sensitivity across states and enable comparison with established Californiabenchmarks, all isolates were tested against five Oomycota fungicides: ethaboxam, mandipropamid, mefenoxam, fluopicolide, and oxathiapiprolin. All isolates were sensitive to the five Oomycota-targeting fungicides evaluated, although significant variation in EC_50_ values occurred within and among states (**Supplementary Table 1**). EC_50_ values were grouped into Scott’s bins and displayed unimodal distributions with no evidence of reduced sensitivity or resistance among isolates from any of the avocado producing regions evaluated (**Figure 4**). Across all isolates, EC_50_ values for ethaboxam ranged from 0.012 µg/ml (PR 5−1 from Puerto Rico) to 0.105 µg/ml (FG 57−2 from California), while mandipropamid EC_50_ values spanned from 0.0018 µg/ml (R8 T1−3 from Florida) to 0.0160 µg/ml (AVO 2−3 from Puerto Rico). For mefenoxam, EC_50_ values ranged between 0.011 µg/ml (LF 2−2 from Texas) and 0.174 µg/ml (AVO 2−3 from Puerto Rico). Fluopicolide displayed a broader range, with EC_50_ values from 0.08 µg/ml (R8 T1−3 from Florida) to 0.61 µg/ml (FG 31−2 from California), representing the least toxic of these five fungicides tested. Oxathiapiprolin exhibited the highest potency, with EC_50_ values from 0.00012 µg/ml (FG 62−1 from California) to 0.00064 µg/ml (AVO 2−3 from Puerto Rico) (**Figure 5**; **Supplementary Table 1**).

Average EC_50_ values differed slightly among states, less than a 1-fold difference for each fungicide, though differences were statistically significant for all fungicides except mandipropamid (**Figure 5**; **Supplementary Table 3**). Across all states, average EC_50_ values remained below 0.036 ug/ml (ethaboxam), 0.007 µg/ml (mandipropamid), 0.055 µg/ml (mefenoxam), 0.37 µg/ml (fluopicolide), and 0.0004 µg/ml (oxathiapiprolin). Among the fungicides, fluopicolide was the least toxic, while oxathiapiprolin was the most potent (**Figure 5**; **Supplementary Table 2**).

### 3.4 Extensive virulence variability observed among U.S. *P. cinnamomi* populations

**Figure 6:**
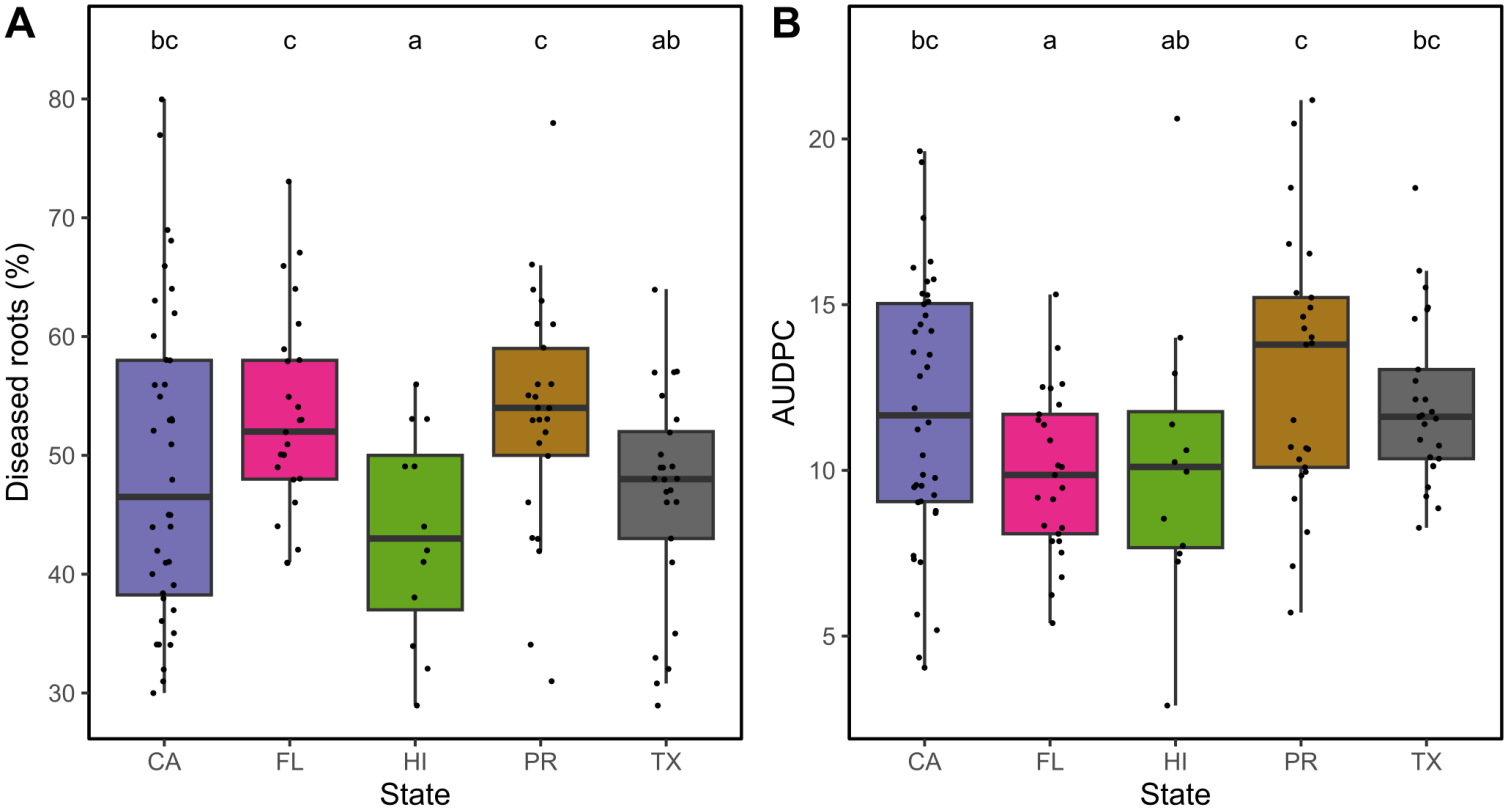
Virulence phenotype of *Phytophthora cinnamomi* isolates assessed in two different hosts. Boxplots showing the median and distribution of **(A)** percentage of diseased roots of UC2001 avocado seedling five-weeks post inoculation with *P. cinnamomi* isolates and **(B)** area under the disease progress curve (AUDPC) values of *P. cinnamomi* inoculated D’Anjou pears. Bars followed by the same letter did not show significant differences in virulence measurements according to the least squared means test at *P* < 0.05. Bars are color-coded based on the geographic origin of isolates (CA = California, FL = Florida, HI = Hawaii, PR = Puerto Rico, and TX = Texas).

Virulence was quantified as percent diseased roots in avocado seedlings and as AUDPC following inoculation of D’Anjou pear fruits. Uninoculated avocado controls remained mostly healthy (94.5%, *data not shown*). Inoculated seedlings exhibited significant differences in disease severity, ranging from 29% to 80% diseased roots, with California isolates spanning the widest range (30% to 80%). The most and least virulent isolates when infecting avocado were from California and Hawaii, respectively (**Figure 6A**; **Supplementary Table 1**). Hawaiian isolates exhibited significant lower virulence than isolates from California,Florida, and Puerto Rico. Consistent with previous findings (Shandset al., 2025), CA−South isolates remained significantly more virulent than CA−North isolates (55.6% vs. 45% diseased roots) on UC2001 seedlings (**Supplementary Table 2**).

Because *P. cinnamomi* can infect a wide range of plants, we also determined if the virulence of these isolates would translate across different hosts and tissue types. To address this, we inoculated D’Anjou fruits pears. We detected significant differences in the AUDPC values amongst all isolates, ranging from 2.9 (HI-5-SZ1) to 21.17 (AVO 1-1), with isolates from Hawaii exhibiting the largest variability (2.9-20.61) followed by those from Puerto Rico (5.71-21.17). On pear fruit, the most virulent isolates originated from Puerto Rico and the least from Hawaii (**Figure 6B**; **Supplementary Table 1**). Isolates from Hawaii differed significantly in AUDPC values when compared to those collected from California, Puerto Rico, and Texas, and unlike the avocado assay, California and Florida isolates differed significantly in their virulence when using pear fruits (**Figure 6B**; **Supplementary Table 3**). In contrast to the avocado assay, no significant differences were detected between CA-South and CA-North isolates on pear fruits (**Supplementary Figure 1**).

## 4 Discussion

We previously documented substantial phenotypic variability in *P. cinnamomi* populations associated with avocado PRR in California (Belisle et al., 2019a, b; Shands et al., 2025). In this study, we expanded that work by characterizing pathogen populations from Florida, Puerto Rico, Hawaii, Texas, and the most current California population for optimal growth temperature, radial growth rate, *in vitro* fungicide sensitivity, and virulence on both D’Anjou pear fruit and moderately resistant UC2001 avocado seedlings. Phenotypic studies of *P. cinnamomi* affecting avocado in the United States have been largely restricted to California (Belisle et al., 2019a, b; Pagliaccia et al., 2013; Shands et al., 2025), while research in other states has focused on pathogen detection and screening for rootstock resistance (Abdulridha et al., 2019; Crandall, 1948; Hwang, 1978; Ploetz et al., 2001; Zentmyer, 1976). Thus, this work represents the first phenotypic characterization of *P. cinnamomi* populations across multiple avocado growing regions in the United States and established *in vitro* baseline sensitivities for three registered (potassium phosphite, mefenoxam, and oxathiapiprolin) and three unregistered (ethaboxam, fluopicolide, and mandipropamid) Oomycota fungicides (Hoyt et al., 2026).

Although *Phytophthora cinnamomi* is heterothallic (A1 and A2 mating types), only clonal A2 populations have been reported in avocado orchards around the world (Dobrowolski et al., 2003; Engelbrecht et al., 2022; Ochoa-Fuentes et al., 2007; Pagliaccia et al., 2013; Shakya et al., 2021; Shands et al., 2025). Shakya et al. (2021) identified two global A2 panglobal clonal lineages (PcG1-A2 and PcG2-A2) and Pagliaccia et al. (2013) described two clonal A2 clades in California (A2 clade I and A2 clade II). Despite the absence of sexual recombination, extensive phenotypic differentiation among clonal populations has been repeatedly documented across hosts and regions (Belisle et al., 2019a, b; Calle-Henao et al., 2020; Hüberli et al., 2001; Ochoa-Fuentes et al., 2015; Shands et al., 2025). The ability to generate large phenotypic variability without sexual reproduction and successfully adapt to climate and control methods highlights the plasticity of this pathogen and demonstrates the challenges for developing effective and durable avocado PRR control methods (Belisle et al., 2019a, b; Calle-Henao et al., 2020; Hüberli et al., 2001; Shands et al., 2025). Consistent with previous observations, in this study, we also detected broad phenotypic diversity for key pathogen traits among isolates from all states evaluated.

Optimal temperature studies from avocado and other hosts have typically reported *P. cinnamomi* optimal growth between 24-28 °C in California (Belisle et al., 2019a; Shands et al., 2025), Mexico (Mondragón-Flores et al., 2022), Australia (Hüberli et al., 2001), and the Pacific Northwest region of the United States (Scagel et al., 2023). In this study, most isolates grew best at 22 °C or 25 °C, with reduced growth at 28 °C. Isolates from Puerto Rico and Texas were particularly adapted to 22 °C, consistent with cooler local climates in collection sites. Similar adaptation to cooler environments has been reported in sub-alpine *P. cinnamomi* populations infecting eucalyptus and avocado (Khaliq et al., 2020). Conversely, we identified isolates from California, Florida, and Hawaii with optimal growth temperatures of 28 or 30 °C (**Table 2**), supporting adaptation to warmer climates. As reported previously (Shands et al., 2025), isolates from CA-South were better adapted to higher temperatures than CA-North isolates, supporting persistent local thermal selection in this state. Higher optimal growth temperatures (>30 °C) have also been reported in other regions (Khaliq et al., 2020). These findings align with the hypothesis that fungal plant pathogens can evolve to acquire thermal stability in response to regional climates (Miñana-Posada et al., 2025), suggesting similar local adaptation within clonal *P. cinnamomi* lineages associated with avocado PRR in the United States.

Excluding potassium phosphite, all Oomycota fungicides exhibited unimodal EC_50_ distributions with narrow ranges across isolates, indicating high baseline sensitivity and low current risk of resistance selection when used appropriately and in rotation programs. Avocado growers rely upon several registered chemicals to control PRR, including phosphonate-based products (e.g., potassium phosphite), phenylamides (e.g., mefenoxam), and only in California, the recently registered piperidinyl thiazole isoxazoline fungicide, oxathiapiprolin (Adaskaveg et al., 2024; Dann and Gazis, 2024). Previous California studies established baseline sensitivities for these compounds and additional Oomycota fungicides (Belisle et al., 2019b). Because comparable data was missing for other avocado producing states, our results provide the first baseline sensitivities for *P. cinnamomi* isolates associated with avocado PRR in Florida, Puerto Rico, Texas, and Hawaii, creating a benchmark for monitoring the emergence or introductions of fungicide-resistant populations.

With the exception of potassium phosphite, the baseline sensitivities of *P. cinnamomi* populations to these fungicides in other states were similar to those previously reported in California, reflecting natural sensitivity levels of this pathogen. As in earlier studies (Belisle et al., 2019a, b), oxathiapiprolin was the most effective fungicide, exhibiting the lowest EC_50_ values. This fungicide also has been shown to be highly toxic to other *Phytophthora* spp. with inhibitory values 10- to 1000-fold lower than those reported for ethaboxam, fluopicolide, mandipropamid, and mefenoxam (Ji and Csinos, 2015; Miao et al., 2016; Qu et al., 2016; Riley et al., 2024). Mefenoxam and metalaxyl have been commonly used to control *P. cinnamomi*, especially in nurseries and natural ecosystems (Dunstan et al., 2008; Hu et al., 2010). Mefenoxam EC_50_ values were consistent with studies in avocado and ornamentals (Belisle et al., 2019a, b; Benson and Grand, 2000; Duan et al., 2008; Hu et al., 2010), reflecting continued effectiveness when used properly.

In contrast, elevated potassium phosphite EC_50_ values, particularly among California and Florida isolates, mirror increasing reports of potassium phosphite-resistant *P. cinnamomi* isolates in California (Adaskaveg et al., 2024; Shands et al., 2025), Australia (Andronis et al., 2024), South Africa (Ma and McLeod, 2014), and New Zealand (Hunter et al., 2023). Similar resistance to this chemical has been documented in *P. cinnamomi* isolates infecting other hosts (Vegh et al., 1985) and in other oomycete pathogens (Brown et al., 2004; Hao et al., 2021). Although most isolates remained sensitive (EC_50_ < 50 µg/ml), significantly higher EC_50_ values were concentrated in regions with long histories of potassium phosphite applications such as California and Florida (Belisle et al., 2019b; Cohen and Coffey, 1986; Mossler and Crane, 2008; Shands et al., 2025). Notably, we reported for the first time, *P. cinnamomi* isolates with EC_50_ values as high as 763.13 µg/ml collected from Santa Barbara. This result is unexpected given previous findings that CA-North isolates (Santa Barbara and Ventura) were more sensitive to potassium phosphite than CA-South populations (Belisle et al., 2019a, b; Shands et al., 2025). This shift may reflect movement of potassium phosphite insensitive isolates from CA-South to CA-North avocado growing regions which is consistent with the recent detection of A2 clade II clonal isolates in CA-North, formerly restricted to CA-South (Santa Barbara) (Pagliaccia et al., 2013; Shands et al., 2025).

In this study, significant variability in pathogen virulence was detected within and acrossstates using avocado and pear fruits inoculation assays. Virulence variability has been previously reported in *P. cinnamomi* across hosts and regions (Ochoa−Fuentes et al., 2015; Robin and Desprez−Loustau, 1998; Serrano and Garbelotto, 2020; Shands et al., 2025; Vitale et al., 2018). Pear fruits have been used to assess pathogenicity and as bait for different *Phytophthora* spp., including *P. cinnamomi* (Sanchez et al., 2019; Shands et al., 2025; Swiecki et al., 2024). In this study, isolates, collected from Texas, Puerto Rico, and California exhibited the highest severity when infecting D’Anjou pear fruit. In addition, isolates from Puerto Rico and California were also the more virulent isolates when infecting avocado seedlings followed by isolates from Florida. Previously, Shands et al. (2025) reported oppositive virulence phenotypes between isolates from CA-North and CA-South when tested in pear fruits versus avocado UC2001seedlings. Here, we did not observe opposite phenotypes between these two assays, however, we found several isolates including the previously characterized isolates Pc2113 and Pc2109 (Shands et al., 2025) as well as FG 90-2, R23 T3-4, and TL 5–2 whose virulence phenotypes were opposite when tested in these two hosts (**Supplementary Table 1**).

The most virulent isolate in avocado was collected from San Diego County, aligning with previousreports of heightened virulence in pathogen population from Southern California growing regions(Belisle et al., 2019a; Shands et al., 2025). Consistent with our prior work, the CA-South isolates in this study remained more virulent on avocado than CA-North isolates (**Supplementary Table 2**) (Belisle et al., 2019a; Shands et al., 2025). Moreover, clonal populations of *P. cinnamomi* associated with avocado PRR in Southern California growing regions have been reported to be more virulent in moderately resistant PRR rootstocks including Dusa^®^ and UC2001 (Belisle et al., 2019a, b; Hoyt et al., 2026; Shands et al., 2025). Virulence variability among *P. cinnamomi* isolates collected from avocado have been also reported in Mexico. Ochoa-Fuentes et al. (2015) found that isolates from Mexico were more virulent in Peribán and Uruapan compared to those collected from Salvador Escalante. Identifying the most virulent *P. cinnamomi* isolates among domestic avocado orchards will aid in creating effective PRR management strategies, including the screening of avocado germplasm to develop rootstocks harboring resistance to these virulent populations and the development of phenotype-specific diagnostic tools that could monitor the introduction and spread of more virulent isolates among these avocado producing regions.

In summary, we detected extensive phenotypic variability within *P. cinnamomi* populations across major U.S. avocado producing regions and documented the presence of highly virulent, potassium phosphite insensitive, and thermally adapted isolates. Previous reports suggested that the large phenotypic variability found in clonal populations *P. cinnamomi* associated with avocado PRR could be attributed to polyploidization, gene expansions, and a bipartite genome architecture that might contribute to a faster adaptation to host resistance, chemicals, and climate (Shands et al., 2024, 2025). Future experiments are needed to determine the molecular basis of this large phenotypic variability. Given the pathogen’s wide host range (>5000 species), virulence assessments across multiple hosts are valuable for both agricultural and natural diversity. These findings have important implications for PRR management and establish a critical foundation for future work linking phenotypic patterns to the genetic diversity of *P. cinnamomi* populations in the United States. Understanding the molecular basis of this pathogen’s phenotypic plasticity and adaptability will be essential for developing sustainable, effective, and durable PRR control strategies to support the competitiveness and long-term viability of the U.S. avocado industry.

## Data Availability

The original contributions presented in the study are included in the article/**Supplementary Material**. Further inquiries can be directed to the corresponding author.

## Supplementary material

Supplementary Figure 1 Boxplots showing the average *in vitro* radial growth per day (RGPD) of *P. cinnamomi* isolates collected from CA–North (orange) and CA–South (purple) at 22 °C, 25 °C, and 28 °C. Letters indicate significant differences across growth temperatures within each avocado growing regions and asterisks (*) indicate significant differences detected for isolates between growing regions at each temperature assayed. Significant differences were calculated using GLMM analyses followed by least squared means test at *P* < 0.05.
