# Supplementary figures and images for: Phytophthora cinnamomi populations collected from avocado in the United States exhibit high adaptive capacity to climate and disease control methods
Source: Front Plant Sci. 2026 Jun 22;17:1838248. doi: 10.3389/fpls.2026.1838248 (PMC13333634; doi:10.3389/fpls.2026.1838248)

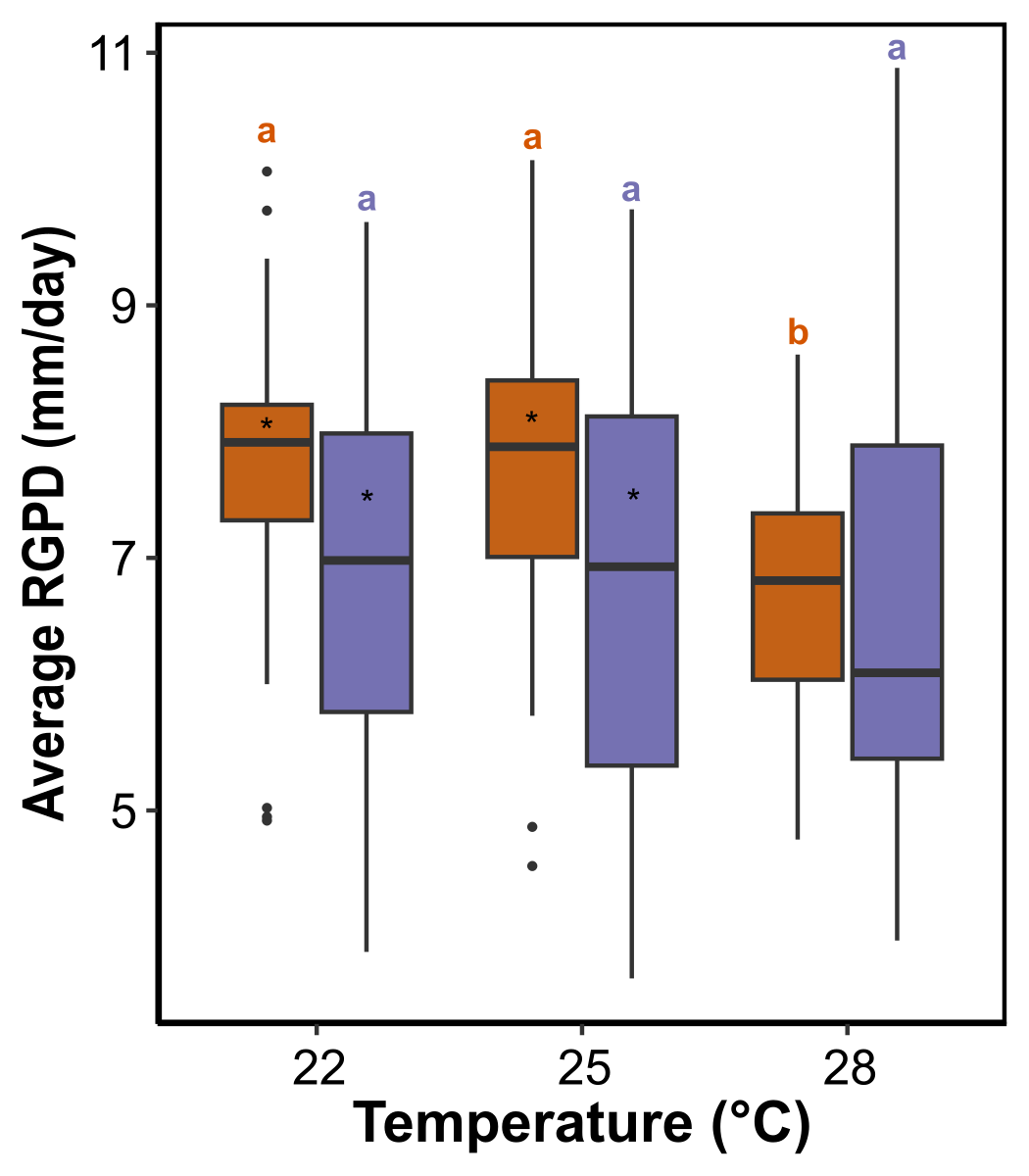

Supplement: Supplementary Figure 1 — Boxplots showing the average in vitro radial growth per day (RGPD) of P. cinnamomi isolates collected from CA–North (orange) and CA–South (purple) at 22 °C, 25 °C, and 28 °C. Letters indicate significant differences across growth temperatures within each avocado growing regions and asterisks (*) indicate significant differences detected for isolates between growing regions at each temperature assayed. Significant differences were calculated using GLMM analyses followed by least squared means test at P < 0.05. [file Image1.tiff]
